# Supplementary material for: Identification of photoperiod-induced specific miRNAs in the adrenal glands of Sunite sheep (Ovis aries)
Source: Front Vet Sci. 2022 Jul 22;9:888207. doi: 10.3389/fvets.2022.888207 (PMC9354845; doi:10.3389/fvets.2022.888207)
Supplement: Supplementary file 1 [file Data_Sheet_1.ZIP › Supplementary file/miRDeep2 file.zip!/miRDeep2 file/Novel_Structure/SPLP42A2/Novel_585.pdf]

Provisional ID	:	NC_019459.2_5355
Score total	:	41.5
Score for star read(s)	:	3.9
Score for read counts	:	34.3
Score for mfe	:	1.7
Score for randfold	:	1.6
Score for cons. seed	:	
Total read count	:	79
Mature read count	:	77
Loop read count	:	0
Star read count	:	2

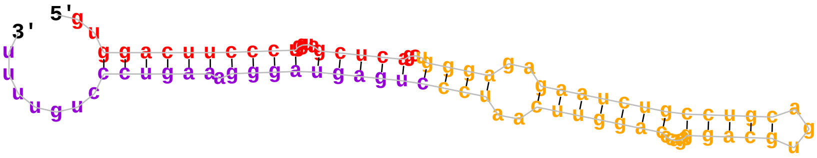

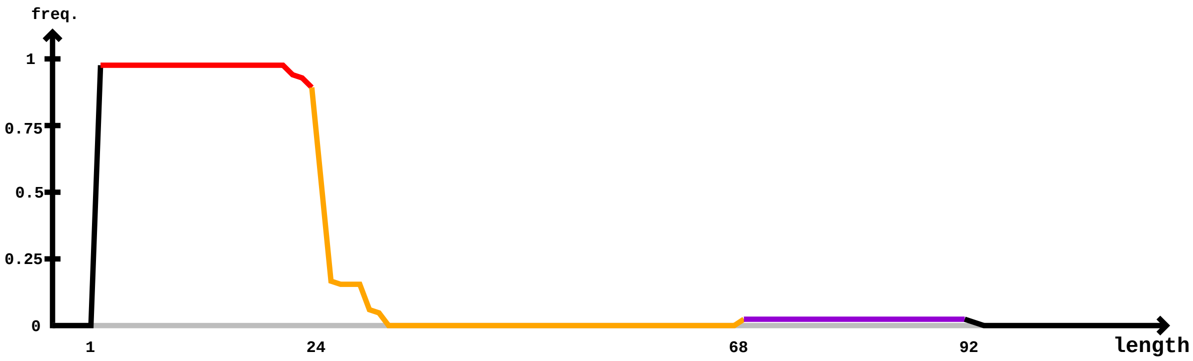

Mature   Star

```
5'- cauguggacuucccugguagcucagcugggagagaaucugccugcagugcaggagacccagguucaaucccugaguagggaaagucccuguuuuuuucucuccccuucuccuuu   -3'    obs
    cauguggacuucccugguagcucagcugggagagaaucugccugcagugcaggagacccagguucaaucccugaguagggaaagucccuguuuuuuucucuccccuucuccuuu          exp
    .....(((((((((....(((((...((((..(((((((((((((...))))).....)))))))..))))))))))))).))))))...........................   reads   mm   sample
    ...guggacuucccugguagcuc...........................................................................................   3       0    seq
    ...gugAacuucccugguagcuca..........................................................................................   1       1    seq
    ...guggacuucccugguagcucag.........................................................................................   3       0    seq
    ...guggacuucccugguagcucagc........................................................................................   47      0    seq
    ...guggacuucccugguagcucagU........................................................................................   13      1    seq
    ...guggGcuucccugguagcucagcu.......................................................................................   1       1    seq
    ...guggacuucccugguagcucagcug......................................................................................   1       0    seq
    ...guggacuucccugguagcucagcuggga...................................................................................   8       0    seq
    ...guggacuucccugguagcucagcugggag..................................................................................   1       0    seq
    ...guggacuucccugguagcucagcugggaga.................................................................................   4       0    seq
    ......................................................................cugaguagggaaagucccuguuuu....................   2       0    seq
```
